# Expanding the repertoire of GalNAc analogues for cell-specific bioorthogonal tagging of glycoproteins†

**DOI:** 10.1039/d4cb00093e

**Published:** 2024-08-22

**Authors:** Abdul Zafar, Sandhya Sridhar, Ganka Bineva-Todd, Anna Cioce, Nadia Abdulla, Vincent Chang, Stacy A. Malaker, David S. Hewings, Benjamin Schumann

**Affiliations:** a Chemical Glycobiology Laboratory, The Francis Crick Institute NW1 1AT London UK; b Department of Chemistry, Imperial College London W12 0BZ London UK b.schumann@imperial.ac.uk; c Tumour-Host Interaction Laboratory, The Francis Crick Institute NW1 1AT London UK; d Department of Chemistry, Yale University CT 06511 New Haven USA; e Vertex Pharmaceuticals (Europe) Ltd., 86-88 Jubilee Avenue, Milton Park Abingdon OX14 4RW Oxfordshire UK

## Abstract

Glycosylation is a ubiquitous modification of proteins, necessitating approaches for its visualization and characterization. Bioorthogonally tagged monosaccharides have been instrumental to this end, offering a chemical view into the cell biology of glycans. Understanding the use of such monosaccharides by cellular biosynthetic pathways has expanded their applicability in cell biology, for instance through the strategy named Bio-Orthogonal Cell-specific TAgging of Glycoproteins (BOCTAG). Here, we show that the cellular use of two azide-tagged analogues of the monosaccharide *N*-acetylgalactosamine (GalNAzMe and GalNPrAz) can be promoted through expression of two biosynthetic enzymes. More precisely, cellular expression of the bacterial kinase NahK and the engineered human pyrophosphorylase AGX1^F383A^ led to biosynthesis of the corresponding activated nucleotide-sugars and subsequent bioorthogonal tagging of the cellular glycoproteome. We explore the use of both sugars for BOCTAG, demonstrating the visualization of cell surface glycosylation tagged with GalNPrAz in a specific cell line in a co-culture system. Our work adds to the toolbox of glycoprotein analysis in biomedicine.

## Abstract 2

Demonstrating the use of azide-containing GalNAc analogues in conjunction with an engineered metabolic pathway to study glycosylation cell-specifically.

## Graphical abstract

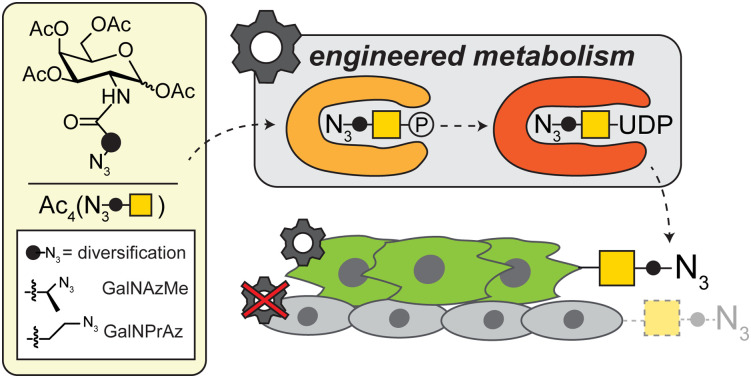

## Introduction

The proteome is substantially expanded through posttranslational modifications (PTMs).^1^ Glycosylation is the most abundant and complex PTM, and tools to understand the glycoproteome are essential to contribute to our understanding in biology. Bioorthogonal chemistry was first developed to study glycosylation due to the lack of genetic methods to directly manipulate glycans. Glycobiology has since played a key role in enhancing bioorthogonal chemistry.

**Fig. 1:**
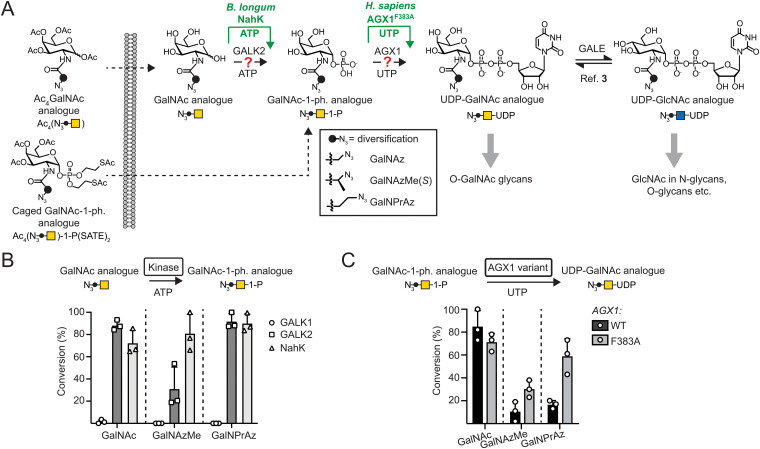
Engineered biosynthesis for UDP-GalNAc analogues. (A) Biosynthetic pathways. UDP-GalNAc is biosynthesized by GALK2 and AGX1 activities, while modifications often require the enzymes NahK and AGX1^F383A^. (B) Acceptance of GalNAc as well as azide-tagged analogues by sugar-1-kinases as measured by UPLC-MS in 16 h reactions. Data are individual data points with means + SD from three independent replicates. (C) Acceptance of GalNAc-1-phosphate as well as azide-tagged analogues by AGX1 constructs as assessed by UPLC. GalNAc-1-phosphate analogues were generated *in situ* by reaction of the corresponding monosaccharides with NahK as shown in (B). Data are individual data points with means + SD from three independent replicates.

A number of monosaccharides have been furnished with chemical tags to allow for bioorthogonal incorporation of reporter compounds such as fluorophores or enrichment reagents. Azide groups were among the first such tags, as they are inert to cellular metabolic processes and are amenable to copper(i)-catalysed (CuAAC) or strain-promoted azide–alkyne (SPAAC) cycloaddition reactions to append reporter groups such as fluorophores.^2–5^ Other tags have included alkynes, alkenes, and many others that are suitable for distinct bioorthogonal reactions.^5–14^ The successful use of tagged monosaccharides relies on their acceptance by biosynthetic enzymes to generate activated sugars, and on glycosyltransferases using these activated sugars as substrates. Appending an azido group to the side chain of *N*-acetylgalactosamine (GalNAc) or *N*-acetylglucosamine (GlcNAc) to render GalNAz or GlcNAz, respectively, allows acceptance by biosynthetic enzymes and incorporation into the glycoproteome.^15–17^ However, even slightly bigger modifications can render monosaccharides refractory to incorporation (Fig. 1A).^3,18–21^

We and others have found that acylamide moieties in GalNAc and GlcNAc with sterically more demanding modifications are often not converted to the corresponding UDP-sugar analogues. In parallel with Chen and colleagues, we have shown that UDP-sugar biosynthesis can be engineered into a cell line for cell-selective incorporation in co-culture systems alongside more advanced *in vivo* systems.^18,21,22^ This strategy, termed Bio-Orthogonal Cell-specific TAgging of Glycoproteins (BOCTAG), employs the bacterial sugar-1-kinase NahK that is more promiscuous towards chemical modifications than the human kinases GALK1 and GALK2.^18,23^ An engineered human pyrophosphorylase AGX1^F383A^ then converts GalNAc-1-phosphates to UDP-GalNAc analogues.^3,19,22,24,25^ Chen and colleagues used the similarly engineered (F383G) pyrophosphorylase AGX2, an isoenzyme to AGX1, along with a bioorthogonal GlcNAc analogue to mediate UDP-sugar biosynthesis.^21^ Both studies employed linear alkynoate side chains as bioorthogonal tags, either a pentynoate side chain *e.g.* in the sugar GalNAlk, or a hexynoate side chain in the sugar GalN6yne. The corresponding UDP-GalNAc analogues are interconverted into their respective UDP-GlcNAc analogues and *vice versa* by the activity of the epimerase GALE, leading to chemical tagging of various sub-types of the glycome. However, only a limited set of sugars have been used to this end. A set of bioorthogonal sugars with alternative chemical tags and potential incorporation into different sub-sections of the glycome would be useful to expand our repertoire of tools to study the glycoproteome.

We have previously identified the bioorthogonal azide-containing GalNAc analogue GalNAzMe to be selectively incorporated into *O*-GalNAc glycans (Fig. 1A).^3^ Selectivity was conferred by the branched nature of the acylamide modification, rendering the corresponding nucleotide-sugar UDP-GalNAzMe resistant to cellular epimerisation by GALE to the respective analogue termed UDP-GlcNAzMe.^3^ In contrast, the nucleotide-sugar UDP-GalNPrAz, an isomer of UDP-GalNAzMe with a linear acylamide, was epimerised to UDP-GlcNPrAz *in vitro*.^3^ While no cellular labelling studies with GalNPrAz were attempted at the time, we reasoned that GalNPrAz may be a more promiscuous metabolic chemical reporter of glycosylation. Testing these hypotheses was challenging at the time since biosynthesis of UDP-GalNAzMe in cells was only possible from a synthetically complex caged sugar-1-phosphate in cells expressing AGX1^F383A^.^3,22^ Our later findings suggesting that the kinase NahK can prime the biosynthesis of chemically tagged UDP-sugars led us to evaluate the metabolic fate of GalNAzMe and GalNPrAz (Fig. 1A). Here, we demonstrate that the BOCTAG principle can be expanded to allow biosynthesis and use of UDP-GalNAzMe and UDP-GalNPrAz from synthetically accessible precursors to increase the variability of metabolic reporters for cell-selective glycoproteome evaluation.

## Results and discussion

### *In vitro* enzymatic synthesis of chemically modified UDP-GalNAc analogues

To anticipate cellular biosynthesis, we investigated how GalNPrAz and GalNAzMe can be enzymatically converted *in vitro* to their respective UDP-GalNAc analogues. We first subjected each monosaccharide alongside GalNAc to recombinant human kinases GALK1, GALK2 or the bacterial kinase NahK and measured turnover to sugar-1-phosphates by Ultra-Performance Liquid Chromatography with Mass Spectrometry detection (UPLC-MS) after either 3 h or 16 h reactions (Fig. 1B and Fig. S1, ESI†). GALK2 accepted both GalNAc and GalNPrAz as substrates to afford >60% and near-complete turnover in 3 h and 16 h reactions, respectively. In contrast, conversion of GalNAzMe by GALK2 proceeded slowly and with approximately 3-times lower conversion after a 16 h reaction. The more promiscuous kinase NahK accepted all three substrates, with >80% conversion of the synthetic compounds GalNAzMe and GalNPrAz after 16 h (Fig. 1B). Similarly, Capicciotti and colleagues observed quantitative phosphorylation of GalNAz by NahK after overnight incubation.^25^ Additionally, acceptance of GalNAz by GALK2 can be inferred from previous findings by us and others that UDP-GalNAz biosynthesis from GalNAz can proceed without expression of a heterologous kinase.^2,16,17^ The galactose-specific kinase GALK1 did not accept any of the three monosaccharides, in line with our previous findings on GalNAc analogues.^18^ We next assessed conversion of sugar-1-phosphates to UDP-sugars by AGX1^F383A^ (Fig. 1C and Fig. S2, ESI†). Monosaccharides were converted to sugar-1-phosphates by NahK *in situ* and treated in a one-pot multienzyme (OPME) reaction with 125 nM WT-AGX1 or AGX1^F383A^. Measuring UDP-sugar biosynthesis by UPLC with UV detection, we found that NahK/WT-AGX1 used chemically modified GalNAc analogues with lower turnover (<20%) compared to GalNAc (91%). In contrast, NahK/AGX1^F383A^ yielded a 67% turnover for UDP-GalNPrAz and 27% turnover for UDP-GalNAzMe in a 16 h reaction (Fig. 1C). The *in vitro* turnover reactions by AGX1 variants matched our previous cellular biosynthesis data.^3^ Reactions stopped after 3 h showed generally low turnover, with the same trends as observed for 16 h reactions (Fig. S2A, ESI†). We tested a higher concentration of 500 nM recombinant AGX1 to corroborate these results (Fig. S2B, ESI†). Including alkyne-tagged GalNAc analogues GalNAlk and GalN6yne in OPME reactions (Fig. S2C, ESI†), we similarly found that NahK together with AGX1^F383A^ produced the corresponding UDP-sugars in near-quantitative turnover in 3 h and 16 h reactions.^18,22,25^ Taken together, the combination of the enzymes NahK/AGX1^F383A^ that featured in our BOCTAG approach successfully synthesised a range of chemically modified UDP-GalNAc analogues from free monosaccharides.

### Cellular biosynthesis of UDP-GalNAzMe and UDP-GalNPrAz

**Fig. 2:**
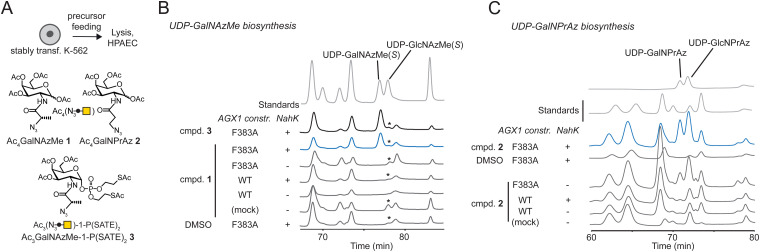
Engineered cellular biosynthesis of UDP-GalNAzMe and UDP-GalNPrAz in K-562 cells. (A) Experimental layout and synthetic compounds used. (B) Biosynthesis of UDP-GalNAzMe in stably transfected cells as measured by HPAEC. Cells were fed with 50 μM synthetic compounds or DMSO. Data are from one representative out of at least two replicates. Asterisk denotes a peak likely due to an artifact of chromatography conditions. (C) Biosynthesis of UDP-GalNPrAz in stably transfected cells as measured by HPAEC. Cells were fed with 50 μM compound 2 or DMSO. Data are from one representative out of at least two independent replicates.

We assessed biosynthesis in stably transfected K-562 cells expressing combinations of NahK and AGX1 constructs. Per-acetylated sugars Ac_4_GalNAzMe 1 and Ac_4_GalNPrAz 2 were used as synthetically convenient monosaccharide precursors (Fig. 2A). A caged GalNAzMe-1-phosphate 3, previously used by us, was employed as a control for biosynthesis.^3^ Mindful that high concentrations of per-acetylated compounds can lead to non-enzymatic background labelling even without UDP-sugar biosynthesis,^26,27^ we included a control cell line transfected with an empty plasmid not encoding either of the biosynthetic enzymes. Cells were fed with sugar precursors, and UDP-sugar biosynthesis was assessed by High Performance Anion Exchange Chromatography (HPAEC) (Fig. 2A and B).^3,19^ Comparison with synthetic standards indicated that cells expressing both NahK and AGX1^F383A^ biosynthesized UDP-GalNAzMe from Ac_4_GalNAzMe 1 and from GalNAzMe-1-phosphate 3 (Fig. 2B). Both NahK and AGX1^F383A^ were needed for UDP-GalNAzMe biosynthesis from Ac_4_GalNAzMe, as cell lines expressing only one of the components or overexpressing WT-AGX1 did not yield a peak at the corresponding retention time (Fig. 2B). Our previous work on the biosynthetic fate of tagged monosaccharides indicated that branched acylamide side chains such as in UDP-GalNAzMe suppress epimerization between UDP-GalNAc analogues and UDP-GlcNAc analogues.^3^ We therefore included the synthetic compound UDP-GlcNAzMe as a standard in our HPAEC experiments.^3^ We could not conclusively rule out epimerisation of UDP-GalNAzMe in these experiments since a peak with variable intensity from cell lysates eluted at the retention time of UDP-GlcNAzMe (Fig. 2B, asterisk). Feeding cells with increasing concentrations of Ac_4_GalNAzMe 1 did not correlate with increasing intensity of this peak (Fig. S3A, ESI†). We therefore concluded that epimerization of UDP-GalNAzMe was not detectable in our assay.^3^ The peak intensity of UDP-GalNAzMe in cells expressing both NahK and AGX1^F383A^ is low (Fig. 2B and Fig. S3A, ESI†), in line with the observed low turnover (27%) of GalNAzMe-1-P to UDP-GalNAzMe by AGX1^F383A^ (Fig. 1C). We ruled out the possibility that endogenous GalNAc may compete with GalNAzMe for phosphorylation since the major pathway for UDP-GalNAc biosynthesis is through epimerization of UDP-GlcNAc by the epimerase GALE.^3,19,28^ In GALE-KO cells, UDP-GalNAc is not detectable, suggesting that levels of free GalNAc are negligible.^3,19^ Lower UDP-GalNAzMe biosynthesis by cells is therefore attributed to less efficient uptake by AGX1^F383A^ (Fig. 1C). UDP-GalNPrAz was successfully biosynthesized in cells expressing NahK and AGX1^F383A^, although small amounts were produced in the absence of NahK (Fig. 2C, and Fig. S3B, ESI†). Including the corresponding UDP-GlcNAc analogue which we termed UDP-GlcNPrAz as a standard, we observed clear epimerization of UDP-GlcNPrAz in cells, although the corresponding peak likewise overlapped with a background peak (Fig. 2C and Fig. S3B, ESI†). Taken together, these data suggested that the presence of the BOCTAG enzymes NahK/AGX1^F383A^ is an efficient way to biosynthesize both UDP-GalNAzMe and UDP-GalNPrAz.

### Incorporation of GalNAzMe and GalNPrAz into cell-surface glycoproteins

**Fig. 3:**
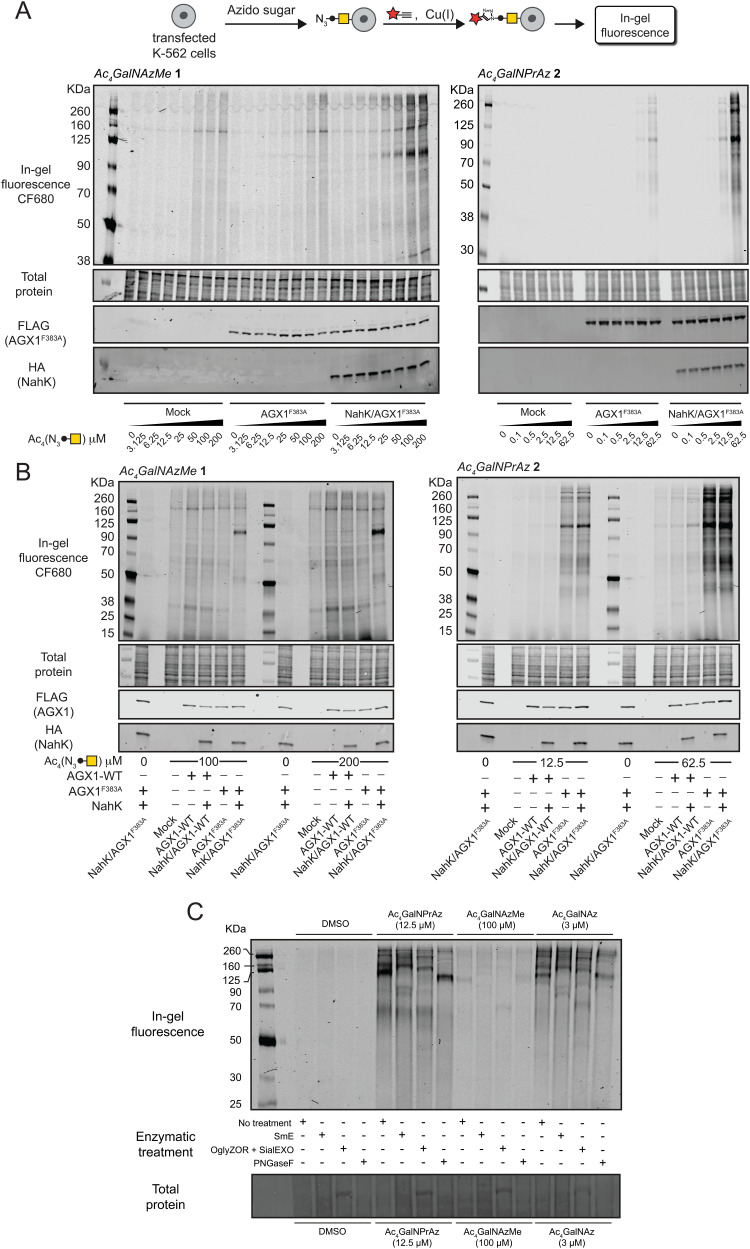
Cell surface incorporation and cell-specific bioorthogonal tagging of azidosugars. (A) Dose-dependent incorporation of GalNAzMe (left) and GalNPrAz (right) into the cell surface glycoproteome of stably transfected K-562 cells as assessed by in-gel fluorescence. Cells were fed with the indicated concentrations of compounds 1 or 2, subjected to cell-surface CuAAC with CF680-alkyne and glycosylation detected by fluorescence scanning. Data are one representative out of two independent replicates. (B) Comparison of stably transfected K-562 cell lines for incorporation of GalNAzMe or GalNPrAz based on optimised feeding concentrations. Data are one representative out of two independent replicates. (C) Comparison of different glycoprotease or glycosidase digestions on lysates of cells expressing both NahK and AGX1^F383A^ fed with per-acetylated GalNAc analogues assessed by in-gel fluorescence.

We assessed incorporation of GalNAc analogues into cellular glycans by on-cell CuAAC with an alkyne-tagged near-infrared fluorophore. This experimental setup ensured that only cell surface glycosylation was detected by ensuing in-gel fluorescence of lysates.^3,19,22,29^ In cells fed with per-acetylated GalNAc analogues Ac_4_GalNAzMe 1 and Ac_4_GalNPrAz 2, both sugar precursors led to a dose-dependent increase in fluorescence signal in cells expressing AGX1^F383A^ and NahK (Fig. 3A). The minimal concentration required for visible signal after Ac_4_GalNAzMe 1 feeding was 25 μM. We observed substantial background fluorescence signal in cell lines fed with Ac_4_GalNAzMe 1, particularly at concentrations above 50 μM. We attributed this signal to non-enzymatic *S*-glyco-modification as a side product seen with per-acetylated bioorthogonal sugars described by Chen and colleagues.^26,27^ However, glycosylation in cells expressing NahK/AGX1^F383A^ led to a clear increase of fluorescence intensity that was specific to individual glycoprotein bands, for instance those at approximately 100 kDa. We attributed this signal to the mucin-like glycoprotein CD43 that is heavily expressed in K-562 cells and tagged by chemically modified GalNAc analogues, as demonstrated previously and herein in an immunoblot co-staining against CD43 (Fig. S4, ESI†).^3,30^ Cells expressing NahK and AGX1^F383A^ showed visible fluorescent signal when fed more than 2.5 μM Ac_4_GalNPrAz 2. In the absence of NahK, feeding with Ac_4_GalNPrAz 2 also led to a dose-dependent fluorescence signal above a minimum concentration of 12.5 μM Ac_4_GalNPrAz 2. Incorporation of GalNPrAz was observed in a much larger range of glycoproteins than GalNAzMe, commensurate with UDP-GalNPrAz being epimerized into UDP-GlcNPrAz that could be used by GlcNAc transferases.^3^ In experiments comparing the labelling intensity of optimal Ac_4_GalNAzMe 1/Ac_4_GalNPrAz 2 concentrations, we further found that overexpression of WT-AGX1 alone or together with NahK does not lead to discernible fluorescence signal (Fig. 3B). Ac_4_GalNAzMe 1 feeding required both NahK and AGX1^F383A^ for bioorthogonal tagging whereas Ac_4_GalNPrAz 2 required at least AGX1^F383A^. These data were corroborated in two other cell lines, the murine 4T1 cancer cell line also expressing green fluorescent protein (4T1-GFP) and the murine MLg fibroblast cell line (Fig. S5, ESI†). Both cell lines incorporated GalNPrAz and GalNAzMe into their glycoproteomes when expressing both NahK and AGX1^F383A^, but not when left untransfected, further confirming that both biosynthetic enzymes lead to reproducible bioorthogonal tagging of the glycoproteome.

The nature of glycoproteins into which GalNPrAz and GalNAzMe had been incorporated was subsequently investigated. Lysates of cells expressing both NahK and AGX1^F383A^ fed with per-acetylated GalNAc analogues were incubated with a panel of glycosidases and glycoproteases (Fig. 3C). The well-characterized, promiscuous GalNAc analogue Ac_4_GalNAz served as a control to compare labelling band patterns by in-gel fluorescence.^16^ GalNAz is a substrate for both the cellular kinase GALK2 as well as NahK as demonstrated previously, both *in vitro* and in living mammalian cells.^16,17,22,25^ The mucinase SmE was recently demonstrated to cleave the protein backbone of mucin-domain glycoproteins with broad tolerance for dense glycosylation and high glycan complexity.^31^ Using SmE, the 100 kDa fluorescent glycoprotein band disappeared across samples fed either Ac_4_GalNAzMe 1, Ac_4_GalNPrAz 2 or Ac_4_GalNAz. A new band appeared at approximately 90 kDa which we attributed to be a digestion product of CD43. A commercial endo-α-*N*-acetylgalactosaminidase (OglyZOR) was next used to remove short core 1 *O*-glycans in the presence of a neuraminidase (SialEXO).^32^ This treatment also led to disappearance of the 100 kDa band across samples fed with all sugars, with the emergence of a new band at approx. 70 kDa that likely lacks untagged *O*-glycans. By using PNGase F that removes N-linked glycans from glycoproteins, the band corresponding to CD43 at approximately 100 kDa remained in all samples, though appeared to be shifted to a slightly lower molecular weight (Ac_4_GalNPrAz 2 treated) or slightly higher molecular weight (Ac_4_GalNAzMe 1 treated). CD43 is known to carry a single *N*-glycan,^30^ the removal of which may impact the charge state of CD43 and thus its migration by electrophoresis. Bands observed at approx. 60, 160 and 260 kDa in cells treated with either Ac_4_GalNPrAz 2 or Ac_4_GalNAz disappeared upon PNGase F treatment, suggesting cleavage of fluorescently labelled *N*-glycans. Previously, we have shown that endowing cells with AGX1^F383A^ and NahK does not appear to induce changes in the glycome or the transcriptome compared to cells transfected with an empty vector.^18^ Therefore, labelling by GalNAz and GalNPrAz reflects naturally occurring glycoproteins. Taken together, these results suggest GalNPrAz and GalNAzMe are indeed incorporated into mucin-type *O*-glycans, and that Ac_4_GalNPrAz 2 recapitulates the labelling of *N*- and *O*-glycoproteins of Ac_4_GalNAz.

### Expanding the monosaccharide repertoire for cell-specific bioorthogonal tagging of glycoproteins

**Fig. 4:**
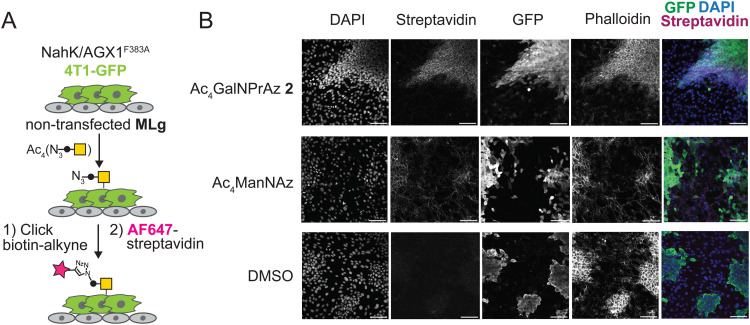
Cell-specific bioorthogonal tagging of glycoproteins with GalNPrAz. (A) Schematic describing set-up of a co-culture system between stably transfected murine 4T1-GFP cancer cells and MLg murine fibroblasts. Cells were fed with compound 2 (62.5 μM), Ac_4_ManNAz (10 μM) or an equivalent volume of DMSO, subjected to on-cell CuAAC with biotin-alkyne, and imaged using fluorescently labelled streptavidin. (B) Microscopy data from one experiment. Scale bar set to 100 μm.

We next tested whether GalNPrAz and GalNAzMe are suitable substrates for BOCTAG, employing a co-culture system between 4T1-GFP and MLg cells.^18^ Initially, 4T1-GFP cells in mono-culture transfected with NahK and AGX1^F383A^ were fed with either Ac_4_GalNAzMe 1, Ac_4_GalNPrAz 2, or the more promiscuous reagent Ac_4_ManNAz that enters the pool of *N*-acetylneuraminic acid.^33^ Incorporation of a clickable fluorophore allowed visualization of cell-surface glycosylation (Fig. S6, ESI†). Cell-surface labelling was clearly observed with Ac_4_GalNPrAz 2 and Ac_4_ManNAz, but not Ac_4_GalNAzMe 1. These data are consistent with our previous finding suggesting that GalNAzMe requires further boosting of signal through engineered glycosyltransferases.^3^ Across samples fed with all of the sugar analogues, artifacts of fluorophore signal not associated with particular cells were observed. (Fig. S6, ESI†) The source of this signal is unknown, but was attributed to a batch effect for fluorescently labelled streptavidin that was not seen in subsequent experiments. As cell-surface labelling was clearly observed with Ac_4_GalNPrAz 2, this sugar analogue was taken forward into a co-culture experiment between non-transfected MLg cells and 4T1-GFP expressing the BOCTAG enzymes. We found that only 4T1-GFP cells expressing the BOCTAG enzymes NahK/AGX1^F383A^ efficiently incorporated GalNPrAz, leading to cell-specific bioorthogonal tagging of glycoproteins (Fig. 4B). In contrast, Ac_4_ManNAz feeding led to ubiquitous cell surface fluorescence on all cell lines. We concluded that Ac_4_GalNPrAz 2 allows for cell-specific bioorthogonal tagging of glycoproteins in the presence of the BOCTAG enzymes NahK/AGX1^F383A^.

## Conclusion

Bioorthogonal tagging techniques have rapidly evolved over the last decades, fuelled by the need to probe the glycome. In recent years, the field has started to map the metabolic fates of bioorthogonal monosaccharides, including their compatibilities with biosynthetic enzymes as well as incorporation into different parts of the glycome. Such metabolic precision is essential to allow the use of bioorthogonal tools in biomedical research. For instance, we have previously shown that GalNAzMe allows investigation of the *O*-GalNAc glycoproteome in a genome-wide genetic knockout screen.^3^ In contrast, GalNPrAz is poised to enter *O*-GalNAc glycans as well as other cellular glycans, presumably *N*-glycans. This finding renders GalNPrAz a more promiscuous tool with higher incorporation efficiency, similar to the alkynoate-containing sugar GalNAlk.^22^

Various strategies have been employed to allow for cell-specific chemical tagging of proteins, including the use of biotin ligases and unnatural amino acids that directly modify the peptide sequence.^34–36^ BOCTAG is complementary to these techniques, targeting glycans as a ubiquitous PTM. Application of the BOCTAG tactic to monosaccharides that enter different glycan subtypes further expands the toolbox for cell-specific labelling of glycoproteins, employing accessible per-acetylated monosaccharide precursors for convenient use in biomedical research.

## Data availability

Data for this manuscript are available in the main text and ESI.† Raw data underlying the manuscript include enzyme assay conversion numbers, raw traces of HPAEC experiments, scanned images of gels and blots, microscopy images, and compound characterization data. These data are securely archived at the Francis Crick Institute and available upon request. Plasmids used herein are available upon request. Synthetic compounds are available upon request as long as stocks last.

## Conflicts of interest

S. A. M. is an inventor on a Stanford patent related to the use of mucinase digestion for glycoproteomic analysis and is a consultant for InterVenn Biosciences.

## Supplementary Material

CB-OLF-D4CB00093E-s001
