# Supplementary material for: Expanding the repertoire of GalNAc analogues for cell-specific bioorthogonal tagging of glycoproteins
Source: RSC Chem Biol. 2024 Aug 22;5(10):1002–9. doi: 10.1039/d4cb00093e (PMC11369666; doi:10.1039/d4cb00093e)

Supplementary Information (SI) for RSC Chemical Biology.
This journal is © The Royal Society of Chemistry 2024

Supporting Information

Expanding the repertoire of GalNAc analogues for cell-specific bioorthogonal tagging of glycoproteins

Abdul Zafar[1,2], Sandhya Sridhar[1,2,3], Ganka Bineva-Todd[2], Anna Cioce[1,2], Nadia Abdulla[1,2], Vincent Chang[4], Stacy A. Malaker[4], David S. Hewings[5], Benjamin Schumann[1,2]*

[1] Department of Chemistry, Imperial College London, W12 0BZ London, United Kingdom.

[2] Chemical Glycobiology Laboratory, The Francis Crick Institute, NW1 1AT London, United Kingdom.

[3] Tumour-Host Interaction Laboratory, The Francis Crick Institute, NW1 1AT London, United Kingdom.

[4] Department of Chemistry, Yale University, CT 06511 New Haven, United States.

[5] Vertex Pharmaceuticals (Europe) Ltd., 86-88 Jubilee Avenue, Milton Park, Abingdon, OX14 4RW Oxfordshire, United Kingdom.

*Correspondence: b.schumann@imperial.ac.uk

Supporting Figures

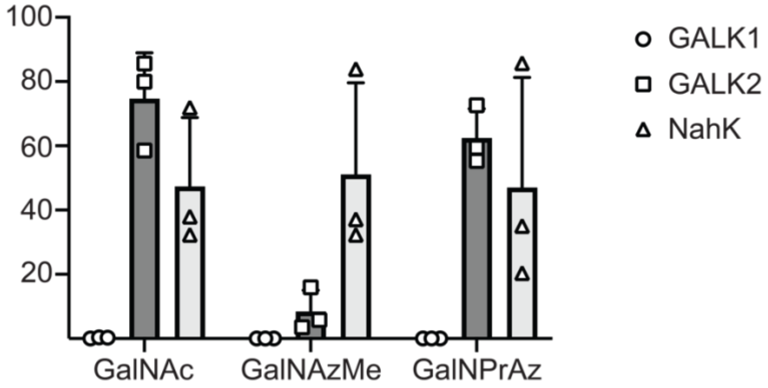

Supporting Fig. 1: Acceptance of GalNAc as well as azide-tagged analogues by sugar-1-kinases as measured by UPLC-MS in 3 h reactions. Data are individual data points with means + SD from three independent replicates.

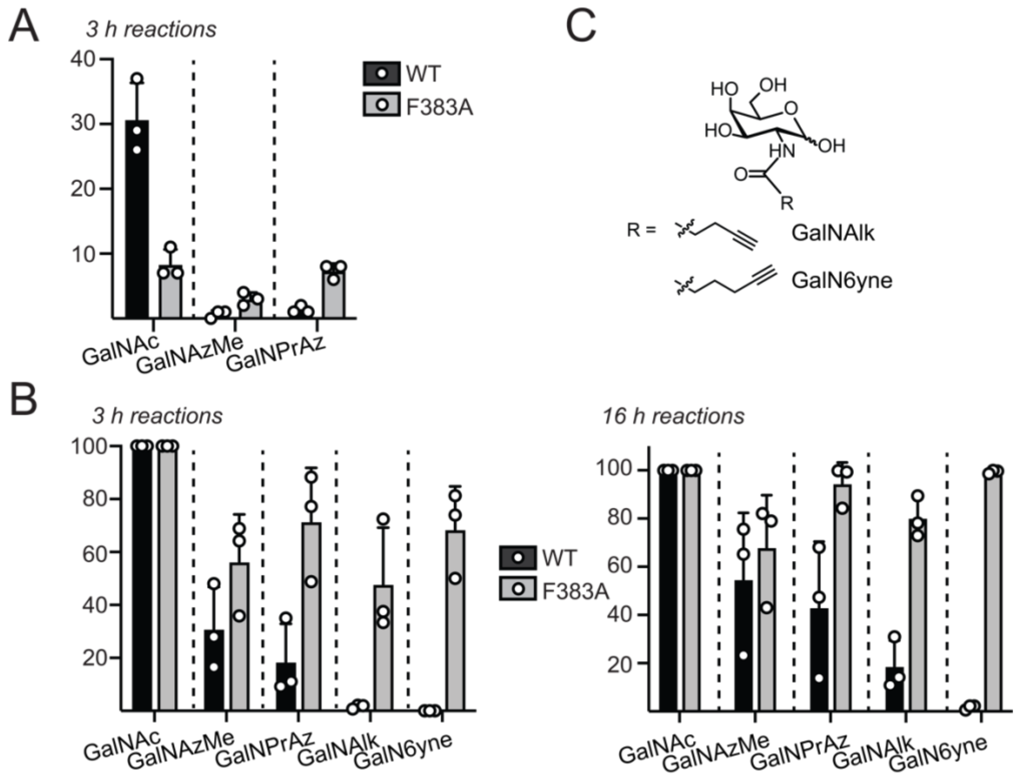

Supporting Fig. 2: Acceptance of GalNAc-1-phosphate as well as azide-and alkyne-tagged analogues by AGX1 constructs (WT and F383A) as assessed by UPLC. GalNAc-1-phosphate analogues were generated *in situ* by reaction of the corresponding monosaccharides with NahK. *A*, 3 h reactions with a concentration of 125 nM recombinant AGX1. *B*, 3 h and 16 h reactions with 500 nM recombinant AGX1. *C*, structures of GalNAlk and GalN6yne. Data are individual data points with means + SD from three independent replicates.

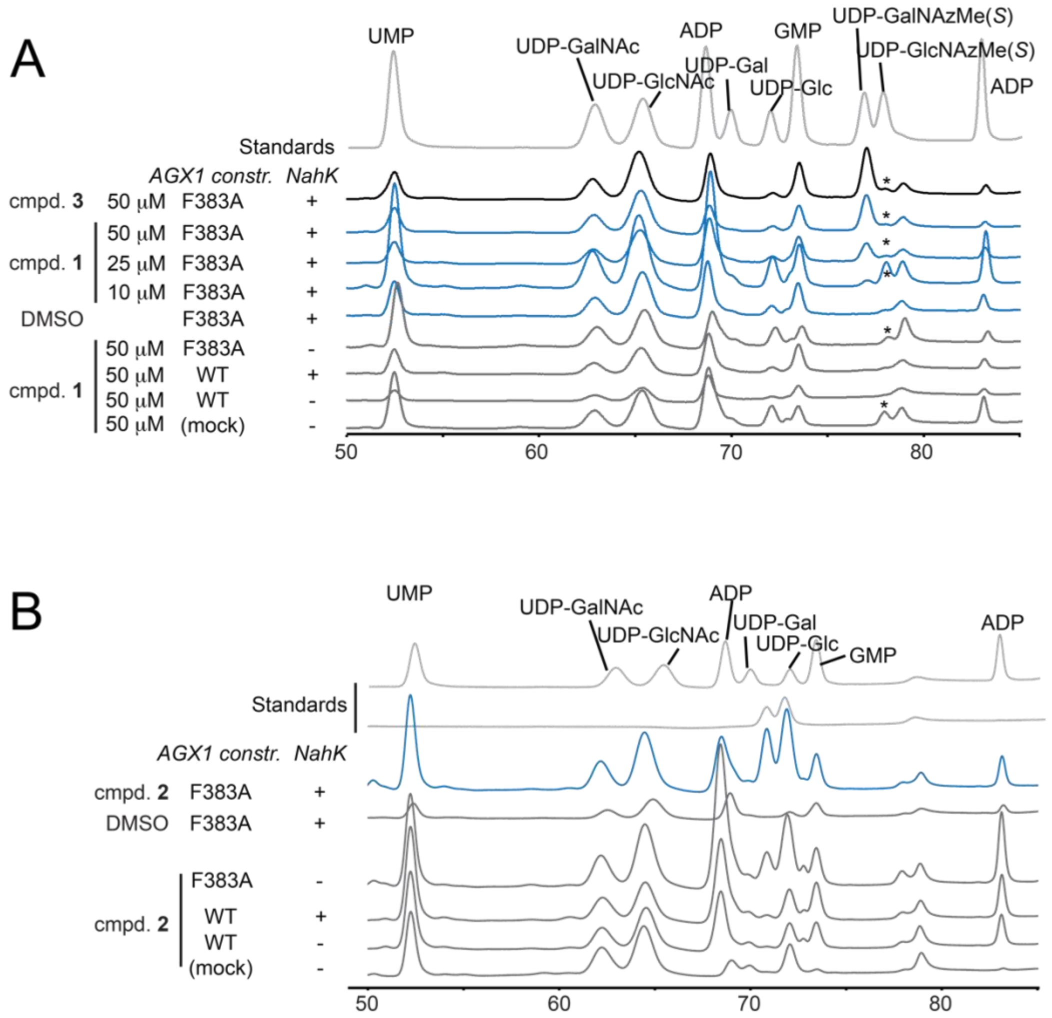

Supporting Fig. 3: Engineered cellular biosynthesis of UDP-GalNAzMe and UDP-GalNPrAz. *A*, biosynthesis of UDP-GalNAzMe in stably transfected cells as measured by HPAEC. Cells were fed with synthetic compounds at the indicated concentrations or DMSO. Data are from one representative out of at least two independent replicates. Asterisk denotes a peak likely due to an artifact of chromatography conditions. *B*, biosynthesis of UDP-GalNPrAz in stably transfected cells as measured by HPAEC. Cells were fed with 50 µM compound **2** or DMSO. Data are from one representative out of at least two independent replicates.

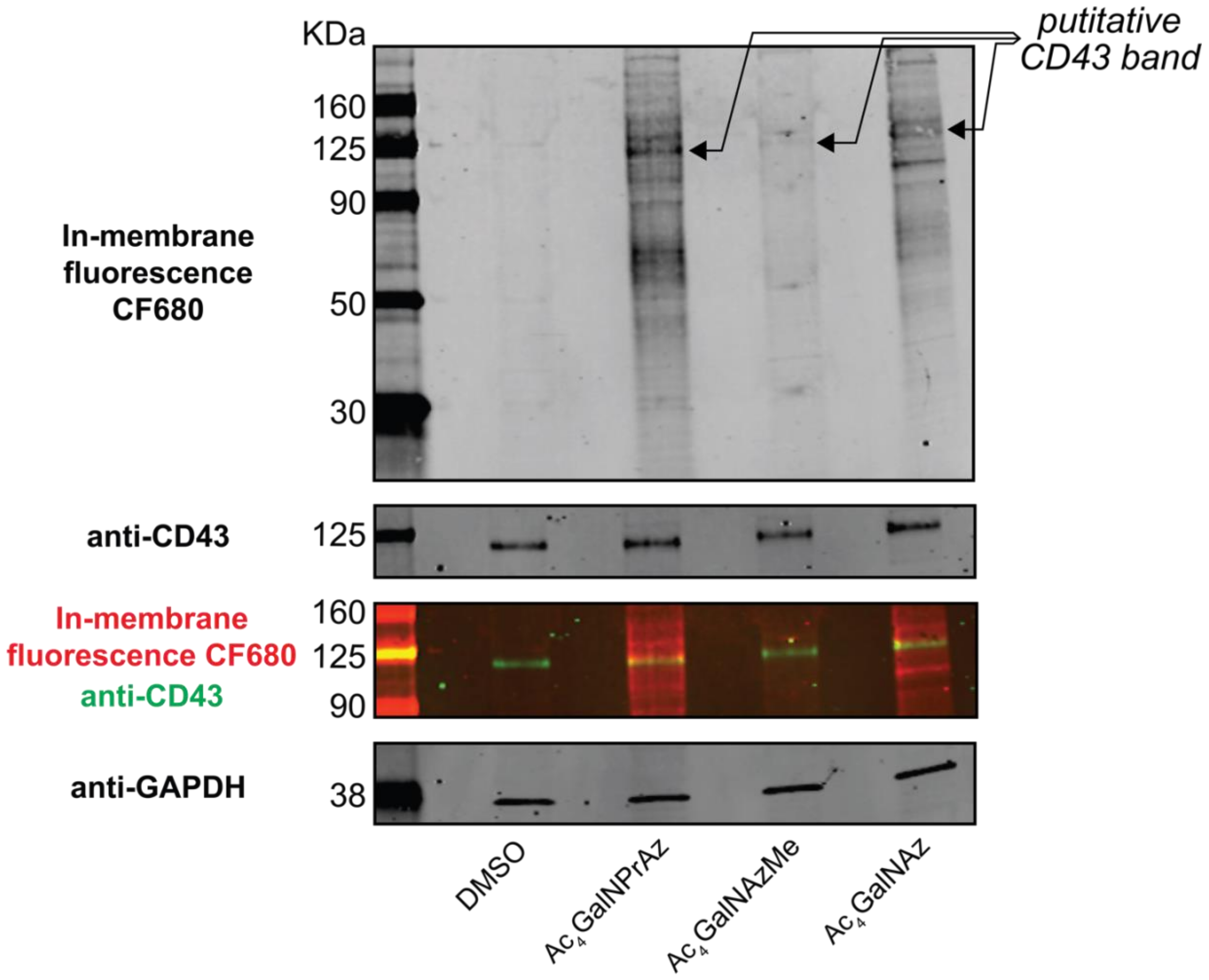

Supporting Fig. 4: Incorporation of GalNAzMe, GalNPrAz and GalNAz in the mucin-domain glycoprotein CD43. K-562 cells expressing AGX1^{F383A} and NahK were subject to overnight feeding with Ac_4GalNAzMe (100 µM), Ac_4GalNPrAz (12.5 µM), Ac_4GalNAz (3 µM) or an equivalent volume of DMSO before cell-surface CuAAC with CF680-alkyne. Cell lysates were run on a polyacrylamide gel and transferred to nitrocellulose, with glycosylation detected by fluorescence scanning and CD43 detected *via* Western blot.

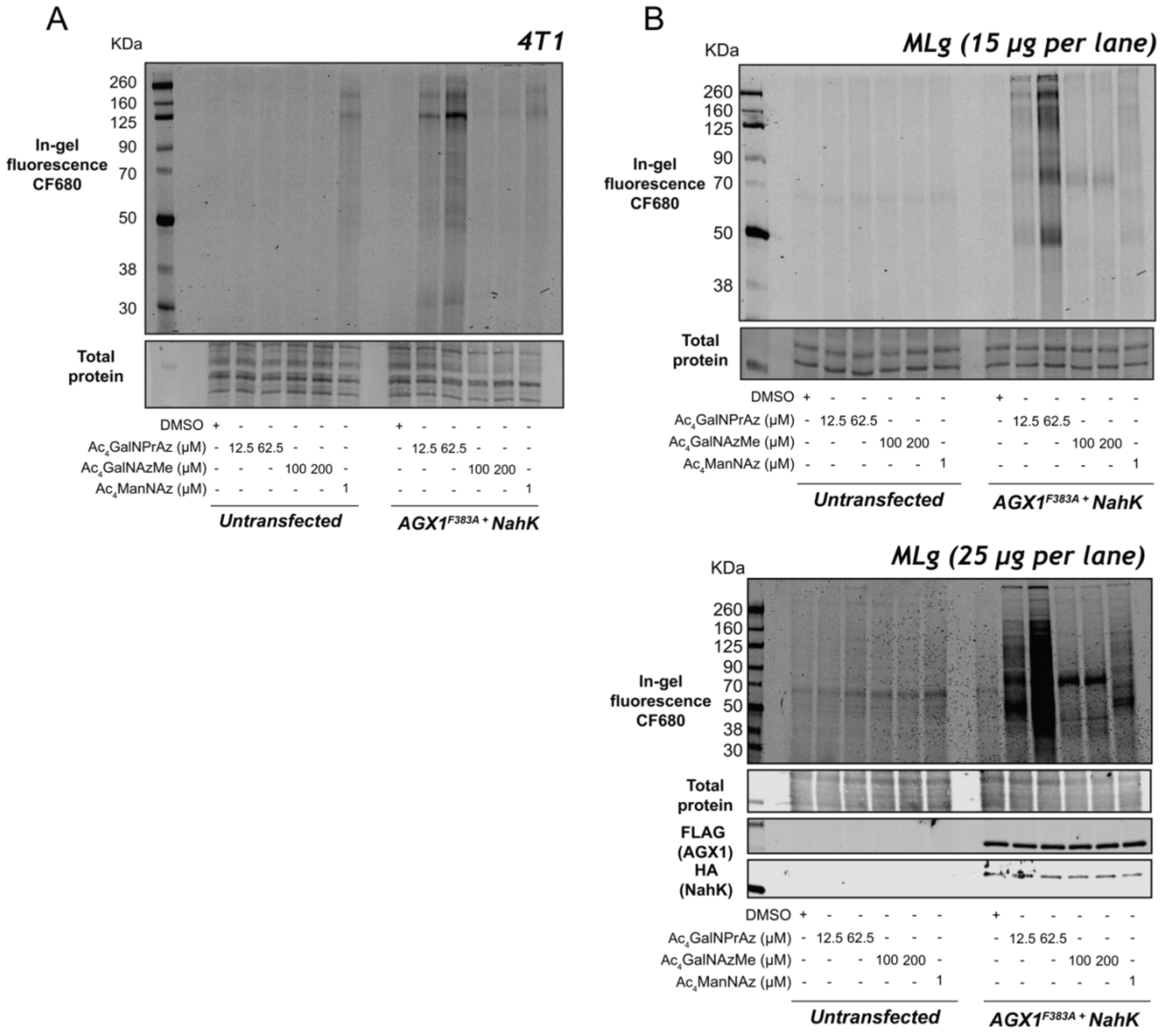

Supporting Fig. 5: Cell surface incorporation and cell-specific bioorthogonal tagging of azidosugars into 4T1-GFP or MLg cells either expressing NahK/AGX1^{F383A} or left untransfected. *A,* 4T1-GFP cells were fed with DMSO or the indicated concentrations of compounds **1**, **2** or Ac$_4$ManNAz, subjected to cell-surface CuAAC with CF680-alkyne and glycosylation detected by fluorescence scanning. Data are from one experiment. *B,* MLg cells were fed with DMSO or the indicated concentrations of compounds **1**, **2** or Ac$_4$ManNAz, subjected to cell-surface CuAAC with CF680-alkyne and glycosylation detected by fluorescence scanning. The same sample was either run on a gel with 15 μg per lane (top) or 25 μg per lane (bottom). Data are from one experiment.

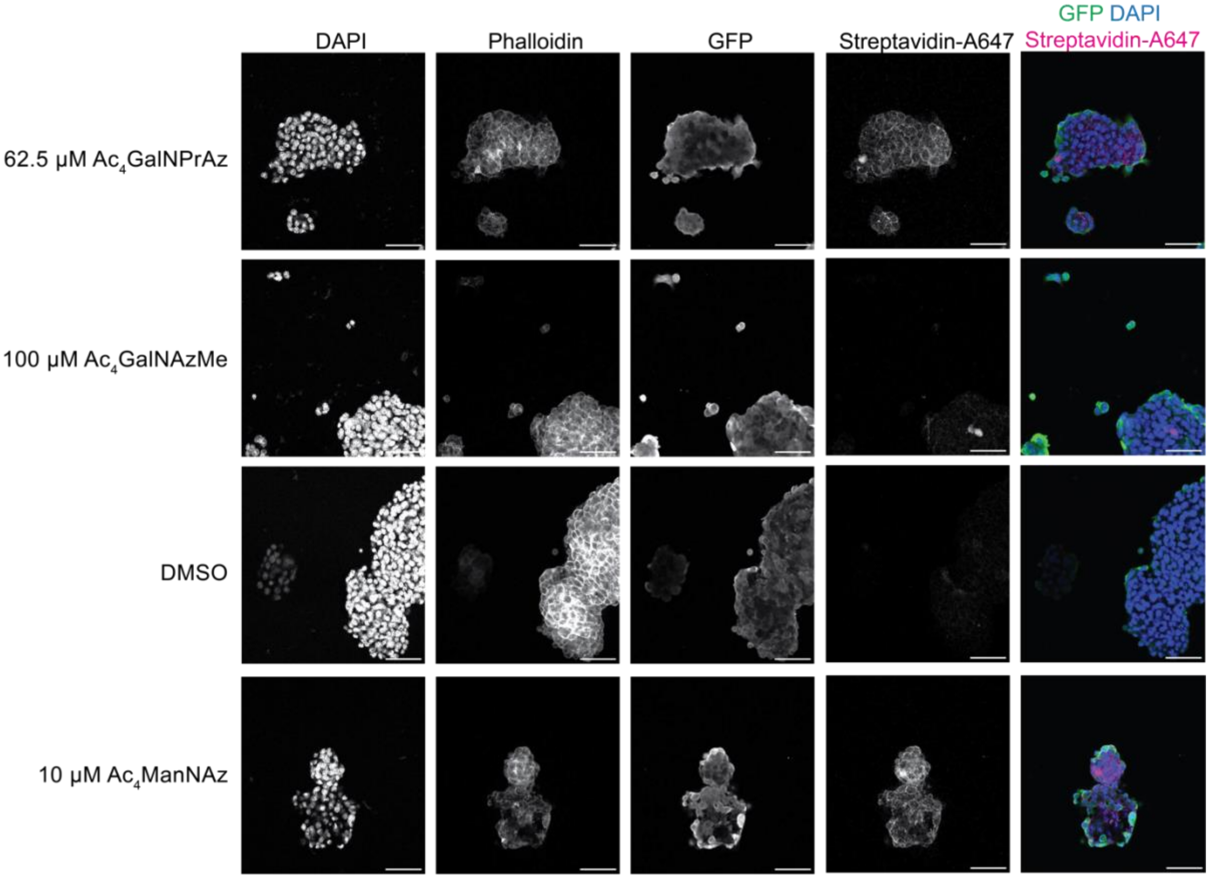

Supporting Fig. 6: Fluorescence microscopy of 4T1-GFP cells expressing NahK/AGX1^{F383A} treated overnight with Ac_4GalNPrAz **2** (62.5 µM), Ac_4GalNAzMe **1** (100 µM), Ac_4ManNAz (10 µM) or an equal volume of DMSO and treated with biotin-picolyl-azide under CuAAC conditions and visualised with AlexaFluor647-Streptavidin. Scale bar, 100 µm. Data are from one experiment.

Experimentals

Synthetic chemistry

Solvents and reagents were of HPLC or synthetic grade. Thin layer chromatography was performed on DC-Fertigfolie Polygram SIL G/UV254 pre-coated with silica polyester sheets (0.2 mm thickness) (Macherey-Nagel, Germany). Spots were developed with sugar stain (0.1% (v/v) 3-methoxyphenol, 2.5% (v/v) sulfuric acid in EtOH) dipping solution. Solvents were removed under reduced pressure using a rotary evaporator and high vacuum. Medium pressure chromatography was performed on an Isolera One flash chromatography system (Biotage, Uppsala, Sweden).

^{1}H and ^{13}C NMR spectra were measured with Bruker Avance-400 MHz spectrometer at 298 K. Chemical shifts (δ) are reported in parts per million (ppm) relative to the respective residual solvent peaks (CDCl3: δ 7.26 in ^{1}H and 77.16 in ^{13}C NMR). The following abbreviations are used to indicate peak multiplicities: s singlet; d doublet; dd doublet of doublets; dt doublet of triplets; m multiplet. Coupling constants (*J*) are reported in Hertz (Hz).

2-(3-azidopropanamido)-2-deoxy-1,3,4,6-tetra-*O*-acetyl-β-D-galactopyranose (2)

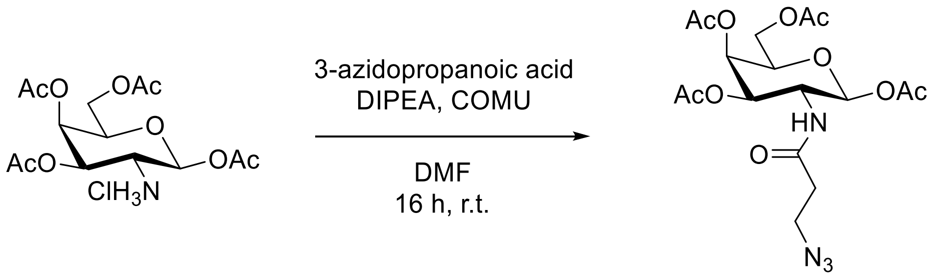

SI-1 **2**

Tetraacetylated glucosamine hydrochloride **SI-1** was prepared by an established procedure.[1] To a stirred solution of 3-azidopropanoic acid (36 mg, 0.31 mmol) in DMF (3 mL) were added amine **SI-1** (100 mg, 0.26 mmol), DIPEA (136 mL, 0.78 mmol) and COMU (134 mg, 0.31 mmol). The solution was left to stir at RT for 16 h. After completion, the reaction mixture was diluted with EtOAc and washed with 0.1 M HCl, sat. $NaHCO_3$ and brine. The organic layer was dried, concentrated, and purified on medium pressure flash chromatography and pre-packed silica column Biotage Sfär Silica D Duo 10 g (cyclohexane: EtOAc; 30-70% EtOAc over 5 CV) to give **2** (61 mg, 0.14 mmol, 54 %, β-anomer only) as a white solid. ^{1}H NMR (400 MHz, $CDCl_3$) δ 5.84 (d, *J* = 9.4 Hz, 1H), 5.76 (d, *J* = 9.0 Hz, 1H), 5.38 (t, *J* = 3.0 Hz, 1H), 5.15 (dd, *J* = 11.3, 3.0 Hz, 1H), 4.44 (q, *J* = 9.8 Hz, 1H), 4.23 – 3.95 (m, 4H), 3.58 (t, *J* = 6.0 Hz, 3H), 2.35 (td, *J* = 6.6, 2.0 Hz, 3H), 2.17 (s, 2H), 2.12 (s, 2H), 2.04 (s, 2H), 2.01 (s, 2H). ^{13}C NMR (100 MHz, $CDCl_3$) δ 170.8, 170.5, 170.4, 170.2, 169.6, 92.8, 71.8, 70.2, 66.4, 61.4, 49.9, 47.2, 35.9, 20.9, 20.7, 20.6.

In vitro enzymatic synthesis of chemically modified GalNAc-1-phosphate analogues

NahK from *B. longum* was purchased from Chemily (Peachtree Corners, USA). Human kinases GALK1 and GALK2 were expressed and purified before.[2]

In vitro reactions for each of the GalNAc, GalNPrAz and GalNAzMe(*S*) sugar analogues were run in 20 µL total volume. Each reaction mixture contained one of the sugars (2.5 mM), ATP (5 mM), $MgCl_2$ (5 mM), Tris-HCl pH 8 (100 mM), BSA (1 mg/mL) and either NahK, GALK1 or GALK2 (4.2 µg). Reactions were run for 3 and 16 hours at 37 °C. At each end point, reactions were stopped by adding equal volume of ice-cold acetonitrile and further cooled on ice for 30 min. The cooled reaction mixtures were centrifugated at 16200 *g* for 30 mins at 4 °C to remove the precipitated enzymes. The supernatants were analysed on UPLC-MS (ACQUITY H-Class qDA, Waters, USA) equipped with ACQUITY UPLC BEH Glycan 1.7 µm 2.1x50 mm column (Waters, USA) and gradient of 90-55% buffer B over 17 minutes at flow rate of 0.35 mL/min and column temperature at 50 °C; buffer A: 10 mM ammonium formate pH 4.5, buffer B: 10 mM ammonium formate 90/10 acetonitrile/water (v/v). Product formation was confirmed by mass detection in negative mode. Turnover (%) was determined by integration of the ion count of the products over the combined ion count of product and remaining substrate. Blanks with the above reaction mixture and no enzymes were included in each set of experiments, treated in the same analysis conditions, and used as a negative control.

In vitro enzymatic synthesis of chemically modified UDP-GalNAc analogues

AGX1 constructs were expressed and purified before.[2] Recombinant inorganic pyrophosphatase PmPpA from *Pasteurella multocida* was purchased from Chemily (Peachtree Corners, USA).

One-pot multienzyme *in vitro* reactions for each of the GalNAc, GalNPrAz and GalNAzMe(*S*) sugar analogues were run in 20 µL total volume. Each reaction mixture contained one of the sugars (2.5 mM), ATP (5 mM), UTP (5 mM), $MgCl_2$ (5 mM), Tris-HCl pH 8 (100 mM), BSA (1 mg/mL), kinase NahK (2.5 µg), PmPpA (0.045U) and either AGX1 WT or AGX1-mut (125 nM). Reactions were run for 3 and 16 hours at 37 °C. At each end point, reactions were stopped by adding equal volume of ice-cold acetonitrile and further cooled on ice for 30 min. The cooled reaction mixtures were centrifugated at 16200 *g* for 30 mins at 4 °C to remove the precipitated enzymes. The supernatants were analysed on UPLC-MS (ACQUITY H-Class qDA, Waters, USA) equipped with ACQUITY UPLC BEH Glycan 1.7 µm 2.1x50 mm column (Waters, USA) and gradient of 90-55% buffer B over 17 minutes at a flow rate of 0.35 mL/min and column temperature at 50 °C; buffer A: 10 mM ammonium formate pH 4.5, buffer B: 10 mM ammonium formate 90/10 acetonitrile/water. Product formation was monitored by absorption at 260 nm and further confirmed by mass detection in negative mode. Turnover (%) was determined by integration of product peaks and plotting against a standard curve of 0-2.5 mM

UDP-GalNAc (Sigma- Aldrich) produced by serial dilution in the final assay buffer. Blanks with the above reaction mixture and no enzymes were included in each set of experiments to account for potential noise signal at the product retention time.

Cell Lines

K-562 and 4T1(GFP) cells stably transfected with pSBbi-GH, pSBbi-AGX1^{WT}, pSBbi-AGX1^{F383A} have been prepared previously.[3] Similarly, K-562 cells stably transfected with pSBbi-AGX1^{WT}-NahK or pSBbi-AGX1^{F383A}-NahK alongside 4T1-GFP and MLg transfected with pSBbi-AGX1^{F383A}-NahK were previously prepared.[2]

All cells were screened for contamination by mycoplasma and other cell lines by the Francis Crick Institute Cell Services Science Technology Platform. All cell culture media and additives were purchased from Thermo Fisher (Waltham, USA). K-562 cells (ATCC CCL-243) were propagated in RPMI containing 10% (v/v) FBS, penicillin (100 U/mL) and streptomycin (100 µg/mL). 4T1 (GFP-expressing, derived from ATCC CRL-2539)[2] and murine MLg fibroblasts (ATCC CCL-206) were maintained in DMEM with 10% (v/v) FBS, penicillin (100 U/mL), streptomycin (100 µg/mL) and GlutaMAX™ Supplement (1X).

Analysis of nucleotide-sugar biosynthesis by High Performance Anion Exchange Chromatography

Compounds **1** and **3** were synthesised previously.[5]
5,000,000 K-562 cells stably transfected with pSBbi-based plasmids were seeded in 10 cm^2 plates in 10 mL of growth media. Cells were incubated overnight (19 h) with 50 µM of either Ac_4GalNAzMe **1**, Ac_4GalNPrAz **2**, Ac_3GalNAzMe-1-P-$(SATE)_2$ **3** or an equivalent volume of DMSO. Following transfer to 15 mL centrifuge tubes, cells were centrifuged (300 *g*, 5 min, 4 °C) and the supernatant was discarded. Cells were resuspended in 1 mL of modified PBS free of Ca^{2+} and Mg^{2+} (Modified PBS; Sigma-Aldrich) and transferred to 1.5 mL microcentrifuge tubes followed by washing twice with Modified PBS. Cell pellets were mixed with a similar volume of 0.1 mm zirconia/silica beads (BioSpec, Bertlesville, USA) alongside 1 mL of a 1:1 (v/v) mixture of ethanol and ultrapure water. Cells were lysed by vortexing (3 x 20 sec) with samples placed on ice for 30 s between each shaking step. Samples were centrifuged (14000 *g,* 10 min, 4 °C), and supernatant transferred to another microcentrifuge tube. Following solvent removal by speed vac, the residue was resuspended in Milli-Q® water (300 µL) and the solution membrane filtered (40 min, 14000 *g*) using a 3 kDa cut-off filter (Merck, Burlington, USA). 100 µL of ultrapure water was added to each filter and centrifuged again (20 min, 14000 *g*). The flow-through was concentrated by SpeedVac followed by resuspension in 60 µL of Milli-Q® water to yield the Cell Lysate.

A 200 µU/µL solution of calf intestinal alkaline phosphatase (CIAP; Thermo Fisher) was prepared according to the manufacturer's instructions. To 20 µL of cell lysate, 1 uL of 200 µU/µL CIAP was added and the reaction was incubated at 37 °C for 30 min. The reaction was quenched by heating to 65 °C for 15 min followed by centrifugation (18000 *g*, 5 min).

The solution was analysed by High Performance Anion Exchange Chromatography using an Arc™ Premier instrument (Waters, Milford, USA) on a Dionex CarboPac

PA1 IC 2x150 mm column (Thermo Fisher) and a 2x50 mm guard column (Thermo Fisher). Solvents were: A = 1 mM NaOH in Milli-Q® water; B= 1 mM NaOH, 1M NaOAc in Milli-Q® water. buffer A= 1M NaOAc/1mM NaOH, B= 1mM NaOH, C= 1M NaOH. 0 min, 5% A, 95% B; 20 min 40% A, 60% B; 60 min, 40% A, 60% B; 63 min, 50% A, 50% B; 87 min, 80% A, 20% B; 95 min, 80% A, 20% B; 96 min, 5% A, 95% B; 101 min, 5% A, 95% B. Commercial or synthetic standards (200-500 μM) were used as controls. Variation in retention time was observed for certain reference peaks across many samples. Chromatographic traces were manually synchronised against a reference spectrum by adding or subtracting time from all values in a spectrum such that a peak for a synthetic or commercial standard was aligned with the corresponding peak in the reference spectrum. Peak picking was performed using HappyTools software.[4]

Metabolic labelling of cell surface glycoproteins for bioorthogonal tagging of azidosugars

K-562 cells (400,000) stably transfected with pSBbi-based plasmids were plated in a 6-well plate in 1.8 mL of growth media. Cells were treated with Ac_4GalNAzMe **1** [5,6] or Ac_4GalNPrAz **2** at the indicated concentrations or corresponding volume of DMSO and grown for 20 h. Cells were harvested (500 *g*, 5 min, 4 °C) in a V-shaped 96-well plate and washed twice with ice-cold 2% (v/v) FBS in PBS (Labelling Buffer, 200 μL). Cells were resuspended in Labelling Buffer (35 μL), followed by treatment with 35 μL of an aqueous solution containing BTTAA (1.2 mM; Click Chemistry Tools, Scottsdale, USA), $CuSO_4 \cdot 5H_2O$ (200 μM; Sigma-Aldrich, St. Louis, USA), sodium ascorbate (5 mM; Thermo Fisher), aminoguanidine hydrochloride (5 mM; Cayman Chemical, Ann Arbor, USA) and CF680 picolyl alkyne (200 μM; Biotium, Fremont, USA). After incubating at RT for 7 min on an orbital shaker, the reaction was quenched by addition of 3 mM bathocuproinedisulfonic acid (BCS) in PBS (35 μL). Cells were harvested (500 *g*, 5 min, 4 °C) and washed four times with ice-cold PBS (200 μL). Cells were then lysed in a solution of 200 μL RIPA buffer (Sigma-Aldrich) containing Benzonase® Nuclease (100 mU/μL; Merck, Rahway, USA) and Halt™ Protease Inhibitor Cocktail (1X; Thermo Fisher) for 20 min at 4 °C with orbital shaking. Lysates were subject to centrifugation (1500 *g*, 20 min, 4 °C) and the supernatant transferred to a new plate. Protein concentration was determined using Pierce™ Rapid Gold BCA Protein Assay Kit (Thermo Fisher) according to the manufacturer's instructions.

A 4x Loading Buffer (a 2:2:1:1 (v/v/v/v) mixture of 1M Tris-Cl pH 6.5, 80% (v/v) glycerol, 20% (w/w) SDS, and 1M dithiothreitol (DTT)) was freshly prepared. 15 μg of protein (unless otherwise specified) were diluted in PBS and 1x Loading Buffer, and the resulting solution was incubated for 5 min. Samples were run on a 10% or 4–20% Criterion™ TGX™ Precast gel (Bio-Rad, Hercules, USA). In-gel fluorescence was detected using an Odyssey® CLx Imaging System (LI-COR Biosciences, Lincoln, USA) before proteins were transferred to nitrocellulose for protein expression assessment. Protein loading was measured using the Revert™ 700 Total Protein Stain Kit according to the manufacturer's instructions except for the Revert 700 Wash Solution which was substituted for an aqueous solution of 6.7% glacial acetic acid and 30% methanol (both from Thermo Fisher). Nitrocellulose membranes were blocked in Intercept® (TBS) Protein-Free Blocking buffer (1 h, RT; LI-COR Biosciences) and antibodies against FLAG tag (rabbit anti-FLAG antibody, 1:1000,

4 °C, O/N, PA1-984B; Invitrogen, Carlsbad, USA) and HA tag (mouse anti-HA antibody, 1:800, 4 °C, O/N, ab18181; Abcam, Cambridge, UK) were employed. All antibodies were diluted in Intercept® T20 (TBS) Antibody Diluent (LI-COR Biosciences) with anti-rabbit secondary antibodies (IRDye® 800CW/700CW Donkey anti-Rabbit IgG Secondary Antibodies, 1:10000, RT, 1 h, 926-32213/926-68073; LI-COR Biosciences) and an anti-mouse secondary antibody (IRDye® 800CW/700CW Donkey anti-Mouse IgG Secondary Antibodies, 1:10000, RT, 1 h, 926-32212; LI-COR Biosciences) employed.

For simultaneous detection of fluorescent CF680 signal and CD43, proteins were transferred to nitrocellulose followed by fluorescence detection as previously described. Nitrocellulose membranes were blocked in 5% BSA (w/v) (Thermo Fisher) in PBS and stained against CD43 (rabbit anti-CD43 antibody, 1:500, RT, 1h, A304-393A; Bethyl Laboratories, Montgomery, USA) and GAPDH (rabbit anti-GAPDH antibody, 1:1000, 4 °C, O/N, ab181602; Abcam). Anti-rabbit secondary antibody was employed as described previously. In-gel fluorescence and Western blot images were processed on Image Studio Lite software (LI-COR Biosciences) and assembled on Illustrator 2021 (Adobe, San Jose, USA).

Metabolic labelling of cell surface glycoproteins for glycosidase and glycoprotease lysate treatment

K-562 cells (400,000) stably transfected with pSBbi-AGX1^{F383A}-NahK were plated in a T25 flask in 5.6 mL of growth media. Cells were treated with $Ac_4GalNAzMe$ **1** (100 µM), $Ac_4GalNPrAz$ **2** (12.5 µM), $Ac_4GalNAz$ (3 µM; Jena Bioscience, Jena, Germany) or a corresponding volume of DMSO, and grown for 20 h. Cells were harvested (500 *g,* 5 min*,* 4 °C) in 15 mL centrifuge tubes followed by resuspension in 800 µL Labelling Buffer and transfered to 1.5 mL microcentrifuge tubes. Cells were washed twice with Labelling Buffer (800 µL). Cells were resuspended in Labelling Buffer (140 µL), followed by treatment with 140 µL of an aqueous solution containing BTTAA (1.2 mM; Click Chemistry Tools), $CuSO_4 \cdot 5H_2O$ (200 µM; Sigma-Aldrich), sodium ascorbate (5 mM; Thermo Fisher), aminoguanidine hydrochloride (5 mM; Cayman Chemical) and CF680 alkyne (200 µM; Biotium). After incubating at RT for 7 min on a vertical rotator, the reaction was quenched by addition of 3 mM bathocuproinedisulfonic acid (BCS; Sigma-Aldrich) in PBS (140 µL). Cells were harvested (500 *g*, 5 min, 4 °C) and washed four times with ice-cold PBS (800 µL). Cells were then lysed in a solution of 800 µL RIPA buffer (Sigma-Aldrich) containing Benzonase® Nuclease (100 mU/µL; Merck) and Halt™ Protease Inhibitor Cocktail (1X; Thermo Fisher) for 20 min at 4 °C with vertical rotating. Lysates were subjected to centrifugation (1500 *g,* 20 min, 4 °C) and the supernatant transferred to new tubes. Protein concentration was determined using Pierce™ Rapid Gold BCA Protein Assay Kit (Thermo Fisher) according to the manufacturer's instructions.

20 µg of protein from cell-surface labelling lysate were diluted to 20 µL in 20 mM Tris-HCl pH 6.8. Samples were either left untreated, or treated with 8 U PNGase F (4 h, 37 °C; Promega, Madison, USA), a mixture of 20 U SialEXO and 20 U OglyZOR (4 h, 37 °C Genovis, Lund, Sweden) or 2 µg mucinase SmE (20 h, 37 °C).[7] 15 µg of protein were diluted in PBS and 1x Loading Buffer, and the resulting solution was incubated for 5 min. SDS-Page, in-gel fluorescence and Western blotting was performed as previously described (Metabolic labelling of cell surface glycoproteins

for cell surface incorporation and cell-specific bioorthogonal tagging of azidosugars investigations).

Fluorescence microscopy

30,000 non-transfected or pSBbi-AGX1^{F383A}-NahK stably transfected MLg cells were seeded into a 24-Well Glass Bottom Plate (Cellvis, Mountain View, USA) in 500 µL growth medium. After 4 h, 3000 4T1 (GFP-expressing) cells were plated on the top of 30,000 MLg cells (1:10 ratio). The co-culture samples were grown for 48 h before feeding with either DMSO, Ac_4GalNAzMe **1**, Ac_4GalNPrAz **2** or Ac_4ManNAz (Jena Bioscience) at the specified concentrations.

Cells were incubated for 16 h. Medium was aspirated, and cells were washed with ice-cold 2% (v/v) FBS in PBS (2 × 200 µL). Cells were then treated with 200 µL of a freshly prepared CuAAC solution containing BTTAA (1200 µM; Click Chemistry Tools), $CuSO_4 \cdot 5H_2O$ (100 µM; Sigma-Aldrich), sodium ascorbate (20 mM; Thermo Fisher), aminoguanidine hydrochloride (20 mM; Cayman Chemical) and Biotin alkyne (200 µM; Biotium) in water. The reaction was carried out for 3 min at RT, the supernatant was aspirated and cells were washed with ice-cold PBS (4 × 200 µL). Cells were incubated for 30 min at RT with 20 µg/mL Streptavidin-AlexaFluor647 (BioLegend UK Ltd, Kentish Town, UK) in 1% (w/v) BSA in PBS solution in the dark. After washing with ice-cold PBS (4 × 200 µL), cells were fixed 20 min with cold 4% (v/v) formaldehyde (Thermo Fisher) in 100 mM sodium phosphate buffer pH 7.4, at RT in the dark. The reaction was quenched by 5 min incubation with 50 mM ammonium chloride and the cells were washed with PBS (3 × 200 µL). Cells were permeabilised with 0.1% (v/v) Triton X-100 in PBS for 10 min at 4 °C and washed with PBS (3 × 200 µL). Cells were blocked in a solution of 10% (v/v) normal donkey serum (ab7475, Abcam), 1% (w/v) BSA and 0.1% (v/v) Tween-20 in PBS for 1 h at RT. GFP expression was detected by incubation with goat anti-GFP (1:300, ab5450, Abcam) in a solution containing 5% (v/v) donkey serum in 1% (w/v) BSA in PBS. Cells were washed with PBS (3 × 200 µL) and incubated for 30 min at RT with AlexaFluor488 anti-goat secondary antibody (1:500, ab150129, Abcam) in a solution containing 1% (v/v) normal donkey serum in 1% (w/v) BSA in PBS. Cells were then incubated with AlexaFluor568 Phalloidin (Invitrogen A12380, 5 µL of 40X methanol stock solution in each 200 µL of PBS) in 1% (w/v) BSA and 0.1% (v/v) Tween-20 followed by PBS washing (3 × 200 µL) and DAPI incubation (1:1000, Vector Laboratories Ltd, Peterborough, UK) in 1% (w/v) BSA and 0.1% (v/v) Tween-20 in PBS for 30 min at RT. After washing with PBS (3 × 200 µL), circle CoverSlips with 15 mm diameter (Thermo Fisher) were mounted onto each well by using 20 µL of ProLong Gold Antifade Mountant (Invitrogen).

The confocal acquisition was made on a LSM710 Invert microscope (Zeiss, Oberkochen, Germany). The images were acquired using a Plan Apochromat 20x/0.8 objective. Samples were imaged with an acquisition zoom of 1.0, so the corresponding resulting pixel size was 0.83x0.83 µM.

A sequential scan to spectrally separate the fluorescence of DAPI, AlexaFluor647, AlexaFluor488 and AlexaFluor568 was used. In addition, the transmitted light channel was activated to visualise the cell morphology. Images were visualised and processed with Fiji and Zen (Zeiss) software.

References

1 N. N. Biswas, T. T. Yu, Ö. Kimyon, S. Nizalapur, C. R. Gardner, M. Manefield, R. Griffith, D. S. C. Black and N. Kumar, *Bioorg. Med. Chem.*, 2017, **25**, 1183–1194.

2 A. Cioce, B. Calle, T. Rizou, S. C. Lowery, V. L. Bridgeman, K. E. Mahoney, A. Marchesi, G. Bineva-Todd, H. Flynn, Z. Li, O. Y. Tastan, C. Roustan, P. Soro-Barrio, M. R. Rafiee, A. Garza-Garcia, A. Antonopoulos, T. M. Wood, T. Keenan, P. Both, K. Huang, F. Parmeggian, A. P. Snijders, M. Skehel, S. Kjær, M. A. Fascione, C. R. Bertozzi, S. M. Haslam, S. L. Flitsch, S. A. Malaker, I. Malanchi and B. Schumann, *Nat. Commun.*, 2022, **13**, 6237.

3 A. Cioce, G. Bineva-Todd, A. J. Agbay, J. Choi, T. M. Wood, M. F. Debets, W. M. Browne, H. L. Douglas, C. Roustan, O. Y. Tastan, S. Kjaer, J. T. Bush, C. R. Bertozzi and B. Schumann, *ACS Chem. Biol.*, 2021, **16**, 1961–1967.

4 B. C. Jansen, L. Hafkenscheid, A. Bondt, R. A. Gardner, J. L. Hendel, M. Wuhrer and D. I. R. Spencer, *PLoS ONE*, 2018, **13**, DOI: 10.1371/journal.pone.0200280.

5 J. Choi, L. J. S. Wagner, S. B. P. E. Timmermans, S. A. Malaker, B. Schumann, M. A. Gray, M. F. Debets, M. Takashima, J. Gehring and C. R. Bertozzi, *J. Am. Chem. Soc.*, 2019, **141**, 13442–13453.

6 M. F. Debets, O. Y. Tastan, S. P. Wisnovsky, S. A. Malaker, N. Angelis, L. K. R. Moeckl, J. Choi, H. Flynn, L. J. S. Wagner, G. Bineva-Todd, A. Antonopoulos, A. Cioce, W. M. Browne, Z. Li, D. C. Briggs, H. L. Douglas, G. T. Hess, A. J. Agbay, C. Roustan, S. Kjaer, S. M. Haslam, A. P. Snijders, M. C. Bassik, W. E. Moerner, V. S. W. Li, C. R. Bertozzi and B. Schumann, *Proc. Natl. Acad. Sci. U. S. A.*, 2020, **117**, 25293–25301.

7 J. Chongsaritsinsuk, A. D. Steigmeyer, K. E. Mahoney, M. A. Rosenfeld, T. M. Lucas, C. M. Smith, A. Li, D. Ince, F. L. Kearns, A. S. Battison, M. A. Hollenhorst, D. Judy Shon, K. H. Tiemeyer, V. Attah, C. Kwon, C. R. Bertozzi, M. J. Ferracane, M. A. Lemmon, R. E. Amaro and S. A. Malaker, *Nat. Commun.*, 2023, **14**, 6169.

Ac4GalNPrAz (2)

^{1}H NMR, $CDCl_3$, 400 MHz

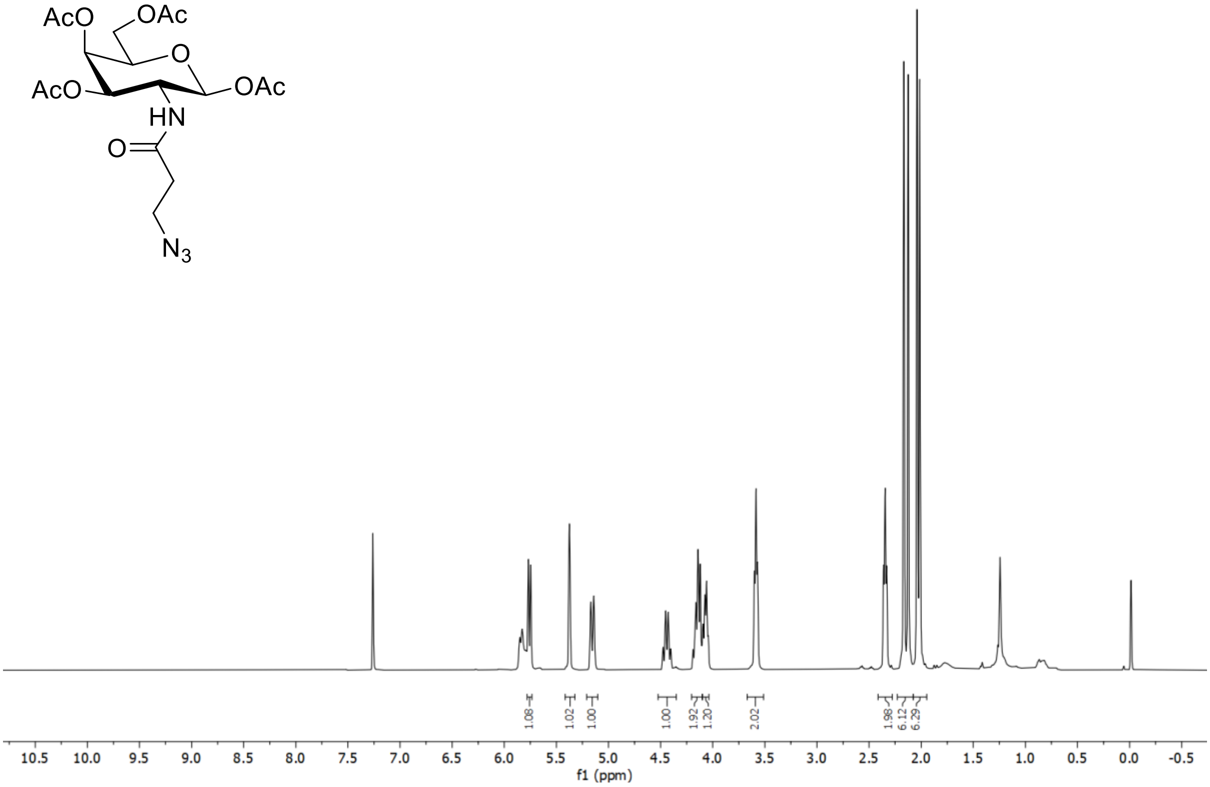

^{13}C NMR, CDCl3, 400 MHz

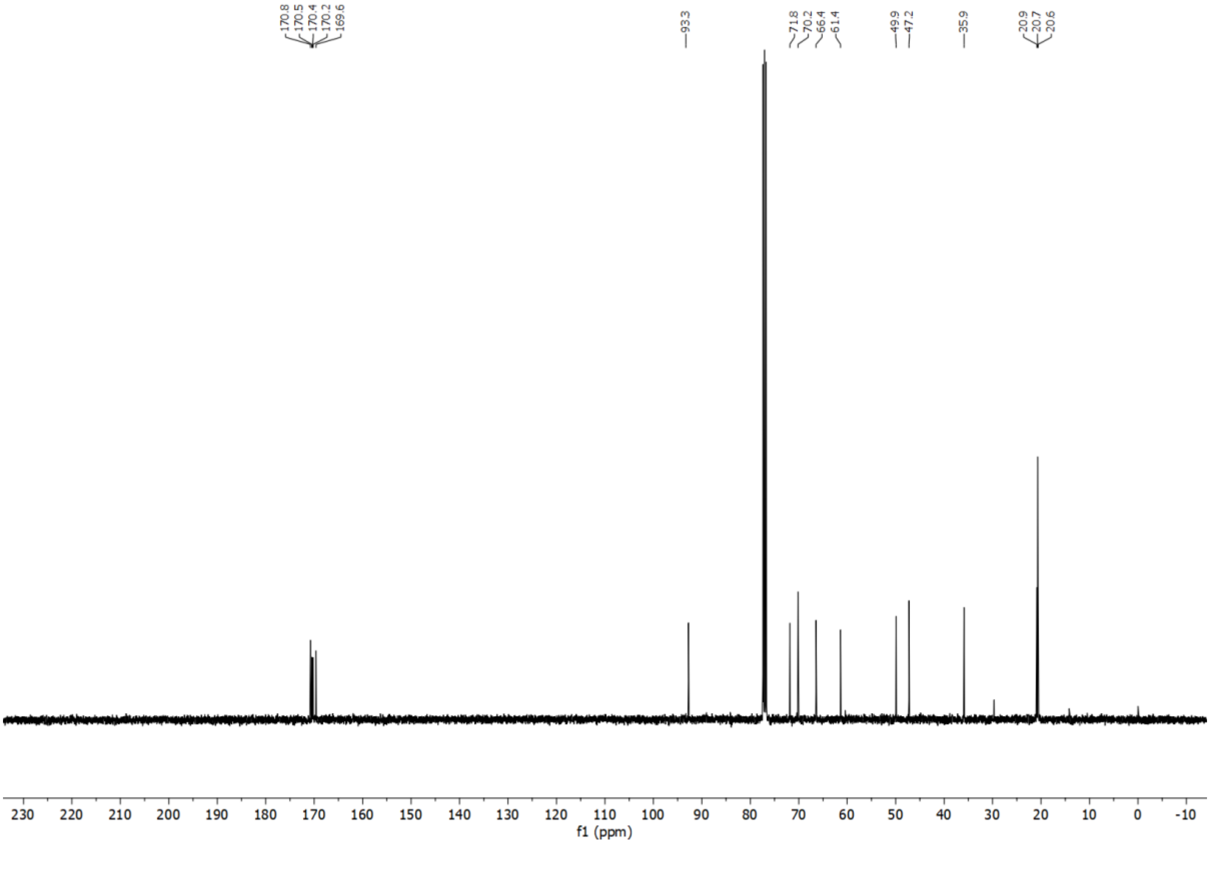

Supplement: CB-005-D4CB00093E-s001 [file CB-005-D4CB00093E-s001.pdf]
